# Potential Use of Extracellular Vesicles Generated by Microbubble-Assisted Ultrasound as Drug Nanocarriers for Cancer Treatment

**DOI:** 10.3390/ijms21083024

**Published:** 2020-04-24

**Authors:** Yuana Yuana, Banuja Balachandran, Kim M. G. van der Wurff-Jacobs, Raymond M. Schiffelers, Chrit T. Moonen

**Affiliations:** 1 Imaging Division, University Medical Center Utrecht, 3584 CX Utrecht, The Netherlands; 2 Department of Biomedical Engineering, TU Eindhoven, 5600 MB Eindhoven, The Netherlands; 3 Department of Pharmaceutical Sciences, University of Utrecht, 3584 CG Utrecht, The Netherlands; 4 Laboratory of Clinical Chemistry and Haematology, University Medical Center Utrecht, 3584 CX Utrecht, The Netherlands

**Keywords:** drug delivery, extracellular vesicles, lysosome, nanocarriers, ultrasound

## Abstract

Extracellular vesicles (EVs)-carrying biomolecules derived from parental cells have achieved substantial scientific interest for their potential use as drug nanocarriers. Ultrasound (US) in combination with microbubbles (MB) have been shown to trigger the release of EVs from cancer cells. In the current study, the use of microbubbles-assisted ultrasound (USMB) to generate EVs containing drug cargo was investigated. The model drug, CellTracker™ green fluorescent dye (CTG) or bovine serum albumin conjugated with fluorescein isothiocyanate (BSA FITC) was loaded into primary human endothelial cells in vitro using USMB. We found that USMB loaded CTG and BSA FITC into human endothelial cells (HUVECs) and triggered the release of EVs containing these compounds in the cell supernatant within 2 h after treatment. The amount of EV released seemed to be correlated with the increase of US acoustic pressure. Co-culturing these EVs resulted in uptake by the recipient tumour cells within 4 h. In conclusion, USMB was able to load the model drugs into endothelial cells and simultaneously trigger the release of EVs-carrying model drugs, highlighting the potential of EVs as drug nanocarriers for future drug delivery in cancer.

## 1. Introduction

A major problem of drug delivery in cancer is that drugs cannot always efficiently bypass the multiple biological barriers. In most of the tissues, the endothelial lining is dense and does not permit the rapid exchange of materials such as drugs between the blood and the interstitial tumour space. Improvements in the drug delivery system to treat cancer including the delivery vehicles may improve the efficacy and safety of drugs [1,2]. 

Nanosized-lipid bilayer vesicles, known as extracellular vesicles (EVs), carry biomolecules such as proteins, lipids and genetic information that originated from the host cells [3]. The nanometre size and targeting modalities enable EVs to penetrate the interstitial tumour space. When EVs are loaded with a drug, this drug cargo can be delivered and transferred to dedicated cancer cells [1,4]. In general, drugs can be loaded into host cells allowing their natural mechanism to package drugs into EVs, also known as endogenous loading [5]. Drugs also can be exogenously loaded into EVs after isolation/purification steps using physical or chemical methods [6]. The feasibility and safety of EVs also have been shown in human clinical trials [7]. Thus, EVs hold potentials as drug nanocarriers.

Ultrasound (US) in combination with gaseous microbubbles (MB), beyond their common use for diagnosis, represents an emerging approach for localized drug delivery [8,9]. Under the action of US waves, MBs transiently affect the permeability of biological barriers (e.g., cell membrane, endothelial lining), thus leading to the uptake and enhanced accumulation of drugs in the region where the US-induced pressure surpasses a certain threshold [10,11]. Despite the potential benefits of USMB-guided drug delivery, the micron size of MB used in this system limits their action exclusively to the vascular bed [12,13]. Thus, future refinements in USMB-guided drug delivery to enhance drug uptake to the targeted tumour cells are warranted. 

Recently, we showed that EVs with a diameter of less than 200 nm were released from head-and-neck cancer cells (FaDu) in vitro for up to 4-fold at 2–4 h after USMB [14]. The fact that USMB triggered a rapid release of EVs shows the significance of this technology to generate EVs and opens opportunities for us to study whether the drug-loaded EVs can be generated following the loading of the drug into the host cells using USMB. 

In this study, we used human endothelial cells (HUVECs) as an in vitro model. CellTracker™ green fluorescent dye (CTG) and bovine serum albumin (BSA) with fluorescein isothiocyanate (FITC) conjugate were used as model drugs. These compounds have distinct characteristics: CTG could freely pass through cell membranes and be transformed into a cell-impermeant fluorescent product in the cytosol, whereas BSA FITC conjugate could pass through cell membranes via endocytosis. Different US acoustic pressures (0.6, 0.7 and 0.8 MPa) were applied to load CTG and BSA FITC into HUVECs. Cells and cell supernatants were harvested and measured to investigate if model drugs were present in the cells and EVs. Subsequently, we highlighted the potential use of EVs as drug nanocarriers by demonstrating the uptake of drug-loaded EVs generated by USMB after 4 h being co-cultured with the recipient cancer cells, FaDu and MDA-MB-231 human breast carcinoma cells. Finally, we utilized the Cre-LoxP system to confirm these findings [15]. 

## 2. Results

### 2.1. Loading Model Drugs into Cells

**Figure 1:**
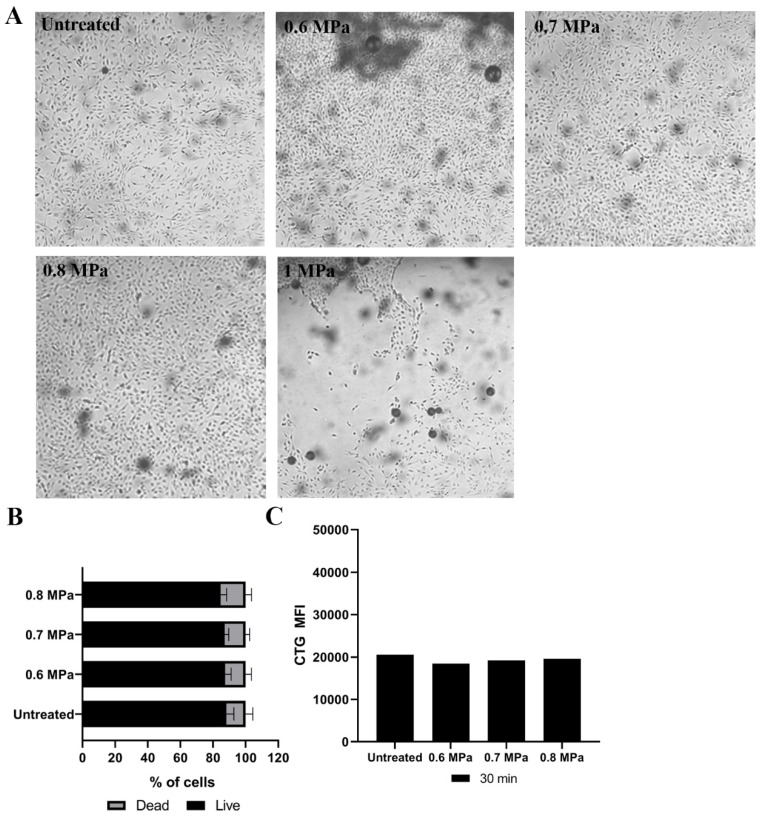
Human endothelial cells (HUVECs) stained with CellTracker™ green fluorescent dye (CTG) and treated at different ultrasound (US) acoustic pressures. Microscopy pictures of HUVECs captured before (untreated) and directly after US treatment (0.6, 0.7, 0.8 and 1 MPa) are presented at 10× magnification (**A**). Afterwards, cells were harvested and stained with PI to determine live and dead cells. The mean percentages of live and dead cells are presented (**B**). US acoustic pressures of 0.6, 0.7 and 0.8 MPa were tested to increase CTG signal intensities in HUVECs after incubation of CTG for 30 min (**C**). Untreated condition was HUVEs after incubation of CTG for 30 min. After 2 h of post-USMB, HUVECs were harvested and measured using flow cytometry (**C**). The mean fluorescent intensities (MFI) of CTG were presented (**C**). As a control, HUVECs without CTG staining were used. MFI results presented are the mean of two independent experiments.

Using the bright-field microscopy, we observed that directly after US application at 0.6, 0.7 and 0.8 MPa, most cells remained attached to the culture chamber (Figure 1A). Increasing the pressure to 1 MPa detached almost 50% of the cells from the surface of the culture chamber, and therefore this condition was not used further in this study (Figure 1A). Untreated and USMB-treated HUVECs (0.6, 0.7 and 0.8 MPa) were harvested and stained using propidium iodide (PI) to determine the percentage of live and dead cells using flow cytometry. The percentages of live and dead cells in treated samples were comparable with the untreated samples (Figure 1B). These results indicated that the US acoustic pressures 0.6, 0.7 and 0.8 MPa could be used safely for loading the model drug into cells in vitro. We observed that HUVECs could be loaded directly with CTG by incubating CTG for 30 min (Figure 1C). However, USMB did not enhance the loading of CTG in HUVECs (Figure 1C). 

**Figure 2:**
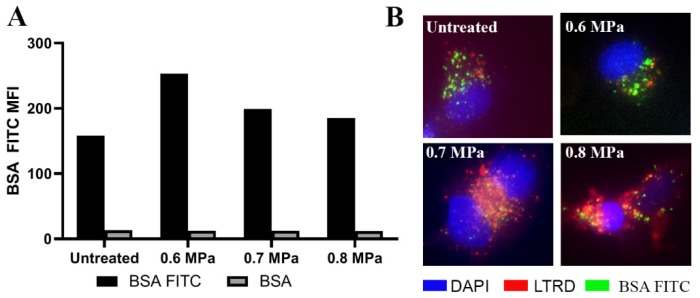
HUVECs loaded with bovine serum albumin conjugated with fluorescein isothiocyanate (BSA FITC) before and after USMB application at different acoustic pressures. After administering BSA FITC with USMB, HUVECs were incubated for 1 h and then, the medium was refreshed. After 2 h, HUVECs were harvested and measured with flow cytometry to quantify the BSA FITC signal, depicted as MFI (**A**). The results presented were the mean of two independent experiments. These cells were also stained with a nuclear stain, DAPI (blue) and a lysosomal marker, LTRD (**B**). Co-localisation of red and green colours indicate the presence of BSA FITC in the lysosomal compartment (**B**). These images were representative images taken at 60× magnification using a fluorescence microscope (Keyence BZ9000, Mechelen, Belgium).

By using USMB, our objective was to increase the delivery of BSA FITC into the cells. We found that loading BSA FITC using different US acoustic pressures was not correlated with the increase of BSA FITC signal in the cells. At 0.6 MPa, BSA FITC signal intensities in HUVECs measured by flow cytometry were the highest compared to untreated and treated HUVECs at 0.7 and 0.8 MPa (Figure 2A). Using a fluorescence microscope, we observed that BSA FITC was present in the HUVECs after treatment with different US acoustic pressures (0.6, 0.7 and 0.8 MPa) and also in the untreated HUVECs (Figure 2B). 

This BSA FITC signal was co-localised with LysoTraker™ Red DND-99 (LTRD) staining in HUVECs, especially after US treatments (Figure 2B). The presence of this molecule in the lysosomal compartment of these cells possibly indicates the degradation of BSA in this compartment. We also co-administered HUVECs with DQ-Red BSA and MB before application of US at 0.6, 0.7 and 0.8 MPa to check if lysosomes were involved in the degradation of BSA. The bright red spots which were indicative for de-quenched fluorescence molecules of DQ-Red BSA when reaching the degradative endo-lysosomes, were detected in HUVECs after US treatment (Figure S1). These red spots were absent in untreated HUVECs (Figure S1). Thus, these results confirmed the presence of BSA in lysosomal compartments after US treatment.

### 2.2. Triggering EV Release Containing Model Drug Cargo 

**Figure 3:**
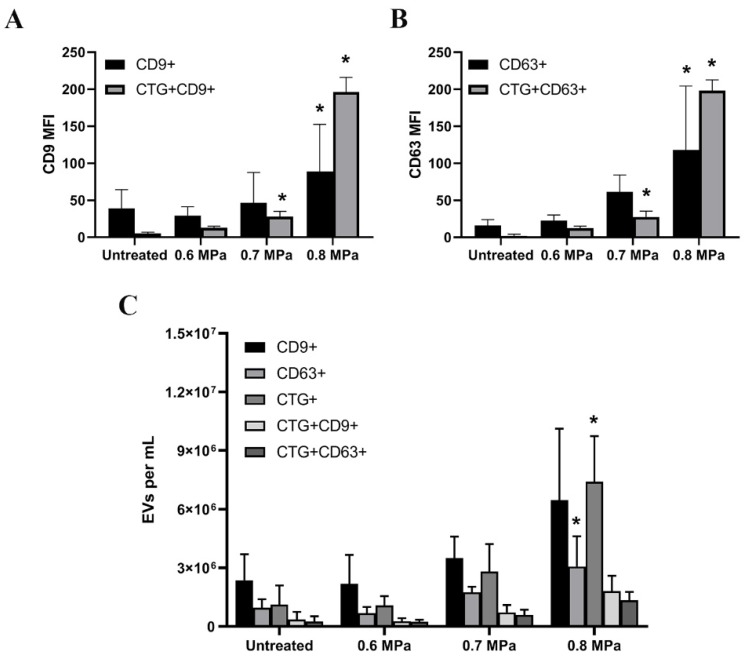
Extracellular vesicles (EVs)-containing supernatants of HUVECs after CTG and USMB treatments measured using flow cytometry. EVs were captured by anti-CD9 and anti-CD63 beads in the supernatants of CTG-stained HUVEC followed by USMB treatment. These EVs were stained with anti-CD9 and anti-CD63 detection antibodies. The MFI of CD9 and CD63 levels in untreated and USMB treated samples (0.6, 0.7 and 0.8 MPa) were quantified using flow cytometry (**A**,**B**). These captured CD9 EVs and CD63 EVs carried CTG (**A**,**B**). Samples were measured in triplicate and these were data from two independent experiments. The same samples were measured using a micro flow cytometer. Anti-CD9 and -CD63 were used to detect EVs bearing CD9 and CD63 antigens present in the cell supernatants (**C**). Total EVs carrying CTG and EVs exposing CD9 or CD63 which carried CTG were also quantified in the same samples (**C**). Results are reported in EVs per mL sample. These were data from two independent experiments. As controls, EVs without CTG were used and all results presented have been corrected from these controls (**A**–**C**). Statistical analysis was performed using one-way ANOVA followed by Tukey’s test. The *p* value < 0.05 was considered significant (*).

Using anti-CD9 and anti-CD63-coated magnetic beads, we captured EVs from the cell supernatants of HUVECs which have been incubated with CTG for 30 min and treated with USMB. USMB triggered an increased release of EVs from HUVEC shown by an increased level of mean fluorescent intensity (MFI) of EVs positive for CD9 and CD63 after USMB treatment especially at 0.8 MPa compared to the untreated, 0.6 and 0.7 MPa conditions (Figure 3A,B). EVs captured by anti-CD9 and anti-CD63 beads carried CTG and the MFI levels of CTG seemed to increase when US acoustic pressures were increased (Figure 3A,B). The measurement of the same cell supernatants using a micro flow cytometer showed that the concentrations of EVs exposing CD9 and CD63 increased 2- and 3-fold subsequently after USMB compared to untreated counterparts, especially at 0.7 and 0.8 MPa (Figure 3C). These EVs also carried CTG and their concentration was significantly higher at 0.8 MPa (*p* < 0.05, Figure 3C). The percentages of EVs positive for CD9 or CD63 which carried CTG generated by USMB at 0.8 MPa are about 20% and 37% respectively.

**Figure 4:**
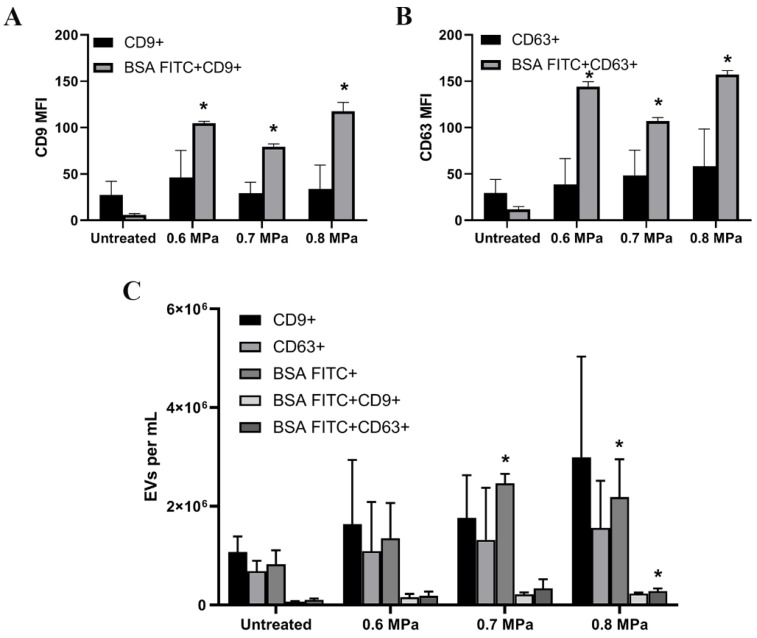
EVs-containing supernatants of HUVECs after co-administration of BSA FITC and MB followed by US treatments measured using flow cytometry. Anti-CD9 and anti-CD63 capture assays coupled to flow cytometry measurement were used to detect CD9 and CD63 EVs present in the conditioned media of HUVEC co-administered with BSA FITC together with MB. Anti-CD9 captured EVs with CD9 on the surface, whereas anti-CD63 captured EVs with CD63 on the surface. Anti-CD9 and anti-CD63 were used as the detection antibodies (**A**,**B**). The MFI of CD9 and CD63 levels in untreated and USMB treated samples (0.6, 0.7 and 0.8 MPa) and also the BSA FITC signals in these captured CD9 EVs and CD63 were quantified (**A**,**B**). Samples were measured in triplicate and these were data from two independent experiments. The same samples were measured by the micro flow cytometer. Anti-CD9 and -CD63 were used to detect EVs bearing CD9 and CD63 antigens present in the conditioned media (**C**). Total EVs carrying BSA-FITC, indicated by EVs exposing CD9 and CD63, which carried BSA-FITC were quantified in the same samples (**C**). Results are reported in EVs per mL sample. These were data from two independent experiments. EVs with BSA were used as controls and all results presented have been corrected from these controls (**A**–**C**). Statistical analysis was performed using one-way ANOVA followed by Tukey’s test. The *p* value < 0.05 was considered significant (*).

**Figure 5:**
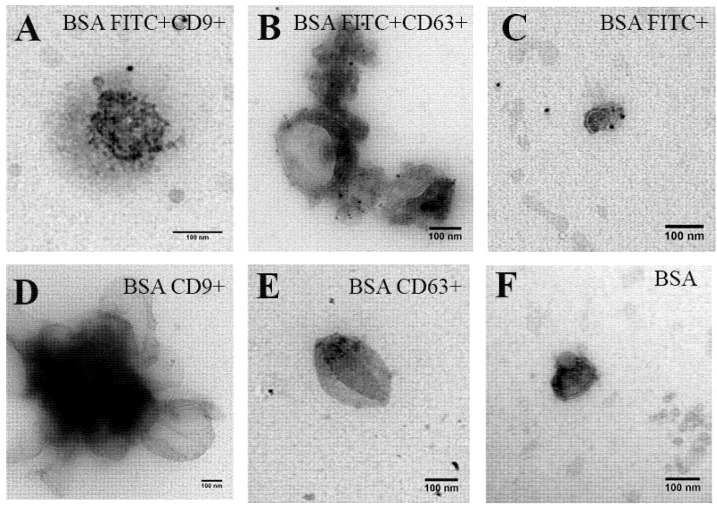
Immunogold electron microscopy of cell supernatant containing EVs derived from HUVEC treated with USMB (0.7 MPa) in the presence of BSA FITC as a model drug. EVs were stained with anti-CD9 or anti-CD63 and the detection antibody was anti mouse IgG secondary antibody labelled with 6-nm gold particles (**A**,**B**). IgG1 isotype control was used as a control for anti-CD9 and anti-CD63 staining (**C**). Anti FITC secondary antibody labelled with 10-nm gold particles stained BSA FITC present on EVS (**A**–**C**). As controls, cell supernatant containing EVs with non-conjugated BSA processed and stained in the same manner (**D**–**F**).

HUVECs loaded with BSA FITC also released EVs containing BSA FITC (Figure 4 and Figure 5). These EVs from the cell supernatants were captured by anti-CD9 and anti-CD63 beads (Figure 4A,B). As controls, BSA was used and EVs present in the supernatant of HUVECs were measured. EVs exposing CD9 and CD63 generated by USMB showed higher BSA FITC fluorescent signal (BSA FITC MFI) than the BSA FITC signal detected from EVs generated by untreated counterparts (Figure 4A,B). However, the increased MFI levels of BSA FITC were not correlated with increasing US pressures. Using the micro flow cytometer, we could detect the concentration of EVs carrying BSA FITC increased significantly at the conditions of 0.7 and 0.8 MPa (2.4 × 10^6^/mL and 2.2 × 10^6^/mL; Figure 4C) compared to the untreated conditions. The percentages of EVs positive for CD9 or CD63 which carried BSA FITC generated by USMB at 0.7 MPA are about 12% and 25% respectively, whereas these generated by USMB at 0.8 MPA are about 10% and 18% respectively. Because of the sensitivity of the micro flow cytometer in measuring single EVs, differences in EV concentrations in condition media of HUVEC treated using different US pressures were detectable. 

To visualise EVs carrying BSA FITC, we also performed immunogold electron microscopy. We used anti-CD9 and anti-CD63 to detect EVs exposing CD9 and CD63 on their surface. Anti-mouse IgG secondary antibody labelled with 6-nm gold particles was used to trace EVs exposing CD9 or CD63. To investigate whether these EVs were carrying BSA FITC, we stained the samples with anti FITC secondary antibody labelled with 10-nm gold particles. We found that CD9-exposing EV carried BSA FITC (Figure 5A). Some CD63-exposing EVs carried BSA FITC (Figure 5B). In Figure 5C, EV carrying BSA FITC was clearly detected. Negative controls were samples containing EVs with BSA (Figure 5D–F). EVs imaged by electron microscopy mostly have a diameter of around 100 nm. This is also confirmed by NTA measurements (Figure S2). These results show that HUVECs which were loaded with BSA FITC released EVs carrying BSA FITC after USMB treatment.

### 2.3. Uptake of EVs Carrying CTG and BSA FITC 

**Figure 6:**
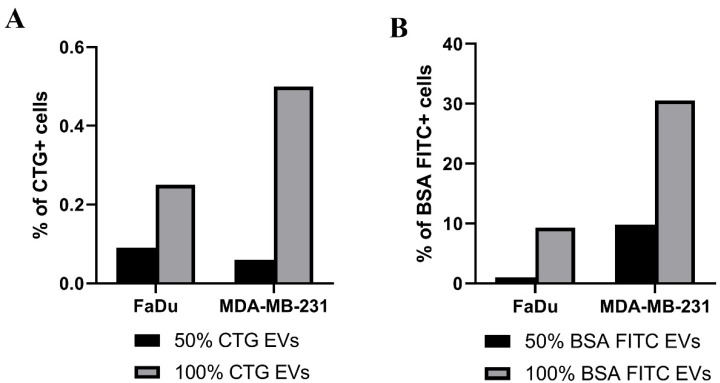
Uptake of EVs containing CTG or BSA-FITC by FaDu and MDA-MB-231 cells. EVs containing CTG (**A**) or BSA-FITC (**B**) at a concentration of 50% and 100% were co-cultured with FaDu or MDA-MB-231 cells for 4 h. The uptake of these EVs was measured by using flow cytometry. Percentages of FaDu and MDA-MB-231 cells exposing CTG or BSA FITC after EV uptake at 4 h-time point are presented (**A**,**B** accordingly). Control conditions were cells co-cultured with EVs which contained no CTG or BSA FITC. Percentages of the positive cells have been corrected for their controls and the uptake of EVs was normalized to 1 × 10^9^ particles/mL.

We performed flow cytometry measurement to quantify the EV uptake by FaDu and MDA-MB-231 after 4 h of co-culturing. The uptake of EV carrying CTG or BSA FITC is shown by the percentages of positive cells for CTG or BSA FITC (Figure 6A,B). After co-culturing with undiluted EVs carrying CTG, there were 0.3% FaDU cells and 0.5% MDA-MB-231 cells positive for CTG (Figure 6A). We also monitored the uptake of EVs containing CTG by MDA-MB-231 cells at 0, 2 and 4 h using a fluorescent microscope (Figure S3). After co-culturing for 4 h, only 2–3 cells per 1.7 cm^2^ were positive for CTG. Thus, the overall results show that the uptake of EVs containing CTG by these cancer cells was low. The same cancer cells took up more EVs carrying BSA FITC (Figure 6B). Particularly, 9.7% of the FaDu cells and 30.5% of the MDA-MB-231 cells were positive after co-culturing with undiluted EVs carrying BSA FITC as detected by flow cytometry (Figure 6B). 

**Figure 7:**
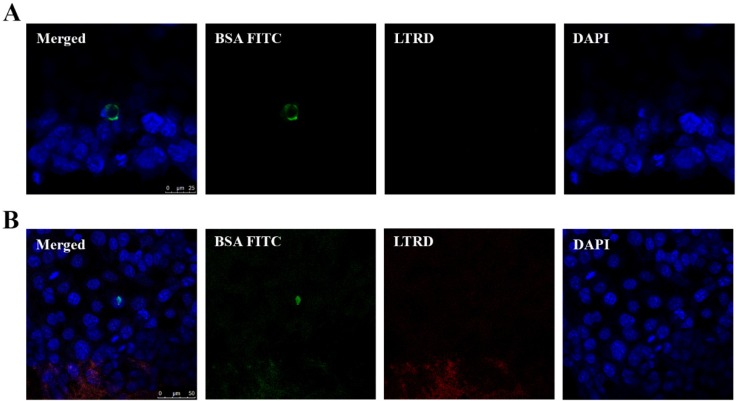
Uptake of EVs containing BSA FITC by FaDu cells. FaDu cells were co-cultured with EVs containing BSA FITC (undiluted). After 4 h, cells were washed with acid wash buffer followed by PBS and fixed prior to staining. LTRD probe (red) was used to stain lysosome and DAPI (blue) to stain the nucleus. Merged images show the uptake of BSA FITC by FaDu cells. BSA FITC signal (green) seems to be present in the cell cytoplasm (**A**, **merged**) or in the vicinity of the cell nucleus (**B**, **merged**).

We also stained the cells with the lysosomal tracker, LTRD, and nucleic acid stain DAPI, to localize the presence of EVs containing BSA FITC in the cells. Using fluorescent and confocal microscopes, we observed that EVs containing BSA FITC were taken up by cells and they were not present in the lysosomal compartment of these cells (Figure 7A,B). The FITC fluorescent signal was observed in the cell cytoplasm or in the vicinity of the cell nucleus (Figure 7A,B accordingly, and Supplementary Video S1).

**Figure 8:**
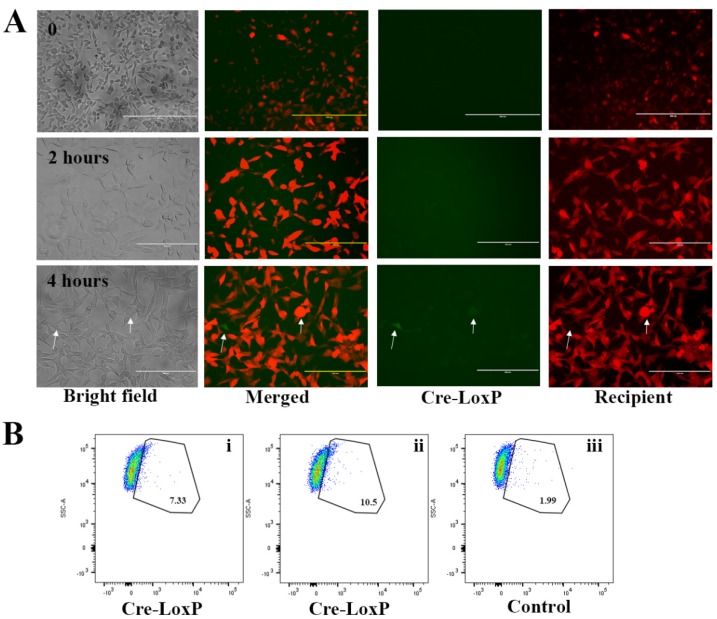
Uptake of EVs carrying Cre recombinase by T47D recipient tumour cells. Undiluted (100%) EVs carrying Cre-recombinase were added to T47D recipient cells (red). At 0, 2 and 4 h, bright field and fluorescent images were taken (**A**). Images were taken at 10× magnification at time point 0. Other images were taken at 20× magnification. Cre-LoxP cells which have a green fluorescent colour were located and shown with a white arrow, both in the bright field and fluorescent images. This uptake was also monitored using flow cytometry. Flow cytometry measurements were performed to assess the presence of Cre-LoxP cells which exposed green fluorescent signal after the co-culturing of EVs carrying Cre recombinase at a concentration of 50% and 100% for 4 h (**Bi**–**ii**). Controls were cells that were co-cultured with EVs without Cre recombinase (**Biii**).

To confirm the uptake of EVs by recipient tumour cells and the release of their cargo in these cells, we co-cultured EVs containing Cre recombinase with T47D receptor cells. The expression of Cre recombinase in EVs has been confirmed by PCR (Figure S4). The colour switch from red to green when receptor cells took up EVs containing Cre recombinase was monitored using a fluorescence microscope and flow cytometer (Figure 8A,B). Using a fluorescent microscope, we monitored the uptake of EVs containing Cre recombinase (100% concentration) at 0, 2 and 4 h (Figure 8A). At 2 h, we could not detect the green Cre-LoxP cells. At 4 h, we located green Cre-LoxP cells (2 cells/0.7 cm^2^ chamber slide, Figure 8A white arrow). Flow cytometry analysis also shows that Cre-LoxP cells were present after co-culturing T47D recipient cells with EVs containing Cre recombinase at a concentration of 50% and 100% (Figure 8Bi,ii). Cells co-cultured with EVs without Cre recombinase served as control (Figure 8Biii). The percentage of green Cre-LoxP cells increased with increasing concentration of EV containing Cre recombinase (7.33% to 10.5%; Figure 8Bi–ii). A red-to-green colour switch observed in reporter-expressing T47D human mammary cells after co-culturing USMB-generated EV carrying active cre recombines confirms the uptake of EVs and transfer of their active cargo into the recipient cells.

## 3. Discussion

In the current study, we observed that increased US acoustic pressure is not always favourable for drug loading. By increasing US acoustic pressure to 1 MPa, cells were already detached from the culture chamber. Lammertink et al. [16] reported that HUVEC detachment was already observed after the application of 0.5 MPa. However, in that study HUVEC was grown on collagen-coated OptiCells^TM^ which may explain this discrepancy. We also noticed that as CTG could freely pass through cell membranes, incubation for 30 min was already sufficient to stain/load HUVECs with CTG and therefore, applying USMB had no additional effect. In the case of BSA FITC loading, applying USMB at 0.6 MPa enhanced the loading. However, increasing US acoustic pressure to 0.7 or 0.8 MPa did not further enhance the loading of this molecule into cells. Based on the degradation of DQ-Red BSA and the de-quenching of the fluorescence after USMB loading, it was likely that our model drug was trapped and degraded in the lysosomal compartment. In conclusion, the type of the model drug is one of parameters which may influence the loading using USMB. It is also clear that this loading would not be optimal if the model drug would be trapped in the lysosomal compartment.

Our previous findings indicated that levels of EVs exposing CD9 or CD63 significantly increased at 2 and 4 h after USMB [14]. Therefore, we measured EVs containing CTG or BSA FITC in the cell supernatant 2 h after USMB treatment. The concentration of EVs containing CTG was 3-fold higher than the concentration of EVs containing BSA FITC, especially when the highest US acoustic pressure was applied (0.8 MPa). This difference may be caused by the mechanism through which these compounds are taken up and sorted by the cells [17]. As CTG could freely enter cell membranes and reside in the cytosol, this compound may avoid endosomal entrapment and perhaps easily be packed into EVs. In contrast, BSA FITC which was internalised by cells via endocytosis may lead to the delivery of endocytosed cargo into the endosomal-lysosomal degradative pathway, influencing the packing of this cargo into EVs [18,19]. It may be necessary to reduce the lysosomal function to prevent the drug compound degradation after internalization and eventually increase the release of EV containing drug [19,20].

Multiple different cellular entry routes are available for EVs and other nanoparticles to cross a cell’s plasma membrane during in vitro and in vivo cell exposure. This cell uptake likely depends on the type of recipient cells and the EV cargo [17,21,22]. In our study, EVs prepared in a similar manner were internalised 2–3-fold more by MDA-MB-231 than FaDu cells. We observed that EVs containing CTG were taken up less than EVs containing BSA FITC. As EVs containing BSA FITC were not observed in the lysosomal compartment indicating that multiple or perhaps independently contributing pathways are involved in EV internalisation processes.

In conclusion, we have demonstrated the feasibility of USMB to load model drugs into endothelial cells allowing the cell’s natural mechanism to generate EVs and package this compound into EVs. Co-culturing these EVs with FaDu and MDA-MB-231 cells resulted in the uptake of EVs and the release of model drug cargo in these cells. These results highlight the potential of EVs as drug nanocarriers for drug delivery.

## 4. Materials and Methods

### 4.1. Cell Culture

HUVECs (pooled donors, Lonza, Verviers, Belgium) were cultured in endothelial cell growth medium 2 with supplement mix (PromoCell GmbH, Heidelberg, Germany). MDA-MB-231 (ATCC^®^ HTB-26™, LGC Standards GmbH, Wesel, Germany) and FaDu (ATCC^®^ HTB-43™) cells were cultured in high glucose Dulbecco’s modified Eagle medium (DMEM) (Sigma-Aldrich, Zwijndrecht, The Netherlands) and 10% foetal bovine serum (FBS) (Sigma-Aldrich). For FaDu cells, this medium was also supplemented with 1% non-essential amino acid (Sigma-Aldrich, Zwijndrecht, The Netherlands). MDA-MB-231 expressing Cre recombinase and T47D receptor cells were a kind gift from Prof. Raymond Schiffelers and these stable cell lines have been generated as described by Zomer et al. [15]. MDA-MB231 cells expressing Cre recombinase were cultured in high glucose DMEM, whereas T47D receptor cells were cultured in DMEM: F12 medium (Lonza, Breda, The Netherlands). In these culture media, 10% FBS, 1% penicillin-streptomycin (Fisher Scientific, Landsmeer, The Netherlands) and 5 μg/mL puromycin (Fisher scientific) were added. All cells were maintained in standard cell culture flasks in a humidified incubator at 37 °C and 5% CO_2_ and passaged twice a week to maintain 80% confluency.

### 4.2. Cell Preparation for USMB

For USMB experiments, cells were prepared according to our previously reported method [14]. Briefly, cells were seeded in cell culture chambers (treated CLINIcell^®^ 175 μm thick polycarbonate walls, 25 cm^2^) (Mabio, Tourcoing, France) 1 day prior to USMB. Media used for maintaining cells in the culture chambers are the same media for maintaining cells in the culture flasks. Except for HUVECs, medium M199 containing L-glutamine (Sigma-Aldrich) and 20% FBS was used. Prior to USMB treatment, these culture media were filtered using 100 KDa centrifugal filter units (Amicon ultra-15, Merck Millipore, Amsterdam, The Netherlands) to deplete EVs related to bovine serum.

### 4.3. Loading Model Drug Using USMB

SonoVue™ (Bracco, Milan, Italy) lipid shelled MB, encapsulating sulphur hexafluoride gas (SF6) were used in the USMB experiments [23]. Microbubbles (MB) were prepared according to the manufacturer’s guidelines.

For co-administrating CTG, cells were first washed with phosphate-buffered saline (PBS) and incubated with 10 nM CTG in serum-free medium for 30 min. As a negative control, cells without CTG were used. Prior to USMB, MB (700 μL) were mixed with 9.5 mL EV-depleted serum-containing medium. This MB-containing medium (final concentration of 0.7–3.5 × 10^8^ MB) was then injected and the cell culture chamber was placed upside down, allowing the MB to rise by floatation towards the cells, ensuring close contact between cells and MB. For co-administrating BSA FITC, cells were first starved for 4 h in serum-free medium before BSA FITC (50 µg/mL) and MB were administered. As a negative control, non-conjugated BSA was administered at the same concentration. We also used DQ-Red BSA (Fisher scientific) to observe the loading of BSA by USMB. Once DQ-Red BSA has reached the degradative endo-lysosomes, it is broken down into smaller fragments which lead to fluorescence de-quenching shown as bright red spots in endo-lysosomes [24], and therefore tracking the location of this molecule in the cell.

Cells and MB were exposed to pulsed ultrasound (10% duty cycle, 1 kHz pulse repetition frequency and 100 μs pulse duration) with varying acoustic pressures at 0.6, 0.7 and 0.8 MPa peak negative pressure (PNP) as was calibrated using a 125 μm glass fibre hydrophone (Precision Acoustics, Dorchester, UK). The piezoelectric unfocused single element transducer with a diameter of 20 mm (Precision Acoustics) operating at 1.5 MHz was placed at the bottom of a water tank, 8 cm below a cell culture chamber frame. The water surface was another 12 cm above the frame and heated to 37 °C. When exposing cells to US, the cell culture chamber was mounted in the frame and moved over the transducer for 80 s to expose the whole cell culture chamber surface, which resulted in a total exposure of approximately 5 s for each area of the cell culture chamber [14,25]. For USMB treatment control, untreated cells were used.

After US exposure, CTG-treated cells were washed carefully with PBS and incubated for another 2 h in EV-depleted serum-containing medium. For BSA FITC-treated cells, we first incubated these cells for 1 h before they were washed carefully with PBS and incubated for another 2 h. After incubation, the conditioned medium was harvested and centrifuged at 300× *g* for 10 min at room temperature (RT) to remove detached cells and large cellular debris. Conditioned medium containing EVs was collected, snap-frozen in liquid nitrogen and stored at −80 °C for EV measurements. Attached cells in the cell culture chamber were detached using trypsin-EDTA solution (Sigma-Aldrich). After serum-containing medium was added, cells were pelleted by centrifuging at 250× *g* for 3 min at RT. To wash cells, cold PBS was added and cells were pelleted again. Finally, cells were re-suspended in 300 µL PBS containing 0.5% BSA and used for flow cytometry measurement.

### 4.4. Flow Cytometry Measurement

The cell suspension was mixed with 5 µL of 10 µg/mL propidium iodide (PI) (Fisher Scientific) and incubated for 5 min. Up to 10,000 events were measured using BD FACScanto II (Becton Dickinson, CA, USA) and the fluorescent intensity of CTG or BSA FITC together with PI was monitored.

For EV measurement using flow cytometry, conditioned medium containing EVs was thawed quickly at 37 °C. We captured EVs using immuno-magnetic beads and measured these EV-bead complexes using BD FACScanto II according to our previously reported method [14]. Using 96-well round-bottom plate 70 µL conditioned medium containing EVs was mixed with 10 µL ExoCap magnetic capture beads CD9 or CD63 (JSR Life Sciences, Leuven, Belgium) to obtain end concentration of 6 × 10^4^ beads per sample. This mixture was incubated overnight at 4 °C with gentle agitation. On the next day, the magnetic plate separator was used to hold EV-bead complexes during washing steps. The supernatant was removed and 100 µL 0.5% BSA-containing PBS (0.45 µm filtered) was added followed by gentle agitation to wash EV-bead complexes for 5 min at RT. After placing the plate on top of the magnetic plate separator, the supernatant was removed. Hundred microliter of diluted mouse anti-human CD9, mouse anti-CD63, or the corresponding mouse IgG1 isotype control coupled to Alexa Fluor 647 were added to the respective wells (see Supplementary Table S1 for the dilutions and manufacturer). This mixture was incubated at RT in the dark for 30 min with gentle agitation. Lastly, the plate was again placed on top of the magnetic plate separator and the antibody/isotype control solution was removed. The plate was washed once with 100 µL 0.5% BSA-containing PBS per well. Samples containing EVs attached to the beads were re-suspended in 250 µL 0.5% BSA/PBS and measured using FACS canto-II. Two independent experiments were performed and from each experiment, samples were measured in triplicate to assess the levels of CD9 and CD63.

The concentration of EVs exposing CD9 or CD63, and EVs carrying CTG or BSA FITC were measured using the A60-Micro flow cytometer (Apogee Flow Systems, Hertfordshire, UK). This flow cytometer enables the measurement of biological particles down to about 100 nm diameter by light scatter [26]. Twenty microliter of conditioned medium containing EVs was incubated with 2.5 µL of mouse-anti human CD9 coupled to PE and 2.5 µL of mouse-anti human CD63 coupled to Alexa Fluor 647. As negative controls, mouse IgG1 isotype controls coupled to PE and to Alexa Fluor 647 were used (please see Supplementary Table S1 for the dilutions of antibodies/isotype controls and their manufacturer). The mixture of samples with antibodies or IgG1 isotype controls were incubated for 15 min and diluted to 225 µL before being measured with flowrate 3.01 µL/ min for 120 s.

### 4.5. Immunogold Electron Microscopy

To investigate whether BSA FITC was carried by EVs after USMB, immunogold electron microscopy was performed as described previously [14,27]. EVs from conditioned medium collected from USMB-treated HUVEC in the presence of BSA FITC or non-conjugated BSA were measured. A thin layer of carbon on the 100 mesh formvar-carbon-coated copper grids (Electron Microscopy Sciences, Hatfield, PA, USA) was evaporated using a carbon-vacuum evaporator according to the manufacturer’s instructions (Edwards Auto 306, West Sussex, UK) just before sample application. Grids were floated directly on top of 10 µL of cell supernatant containing EVs and incubated for 7 min at RT. The grids were washed three times using PBS (0.22 µm filtered). Next, the grids were incubated in a blocking solution (Aurion, Wageningen, The Netherlands) for 30 min at RT followed by washing three times with 0.1% BSA-c (Aurion). For double immuno-labelling, the grids were incubated overnight at 4 °C in a mixture of 20 µL of mouse anti-human primary antibody (anti-CD9 or anti-CD63 antibody). For negative controls, mouse IgG1 isotype control was used. Unbound antibodies were removed from the grids in six washing steps by placing the grids on top of 0.1% BSA-c solution. Afterwards, the grids were incubated with 10 µL goat anti mouse IgG secondary antibody labelled with 6-nm gold particles (Aurion) for 1 h at RT. Grids were washed six times with 0.1% BSA-c before incubation with 10 µL mouse anti-FITC labelled with 10-nm gold particles for 1 h at RT. Dilutions of the antibodies, isotype control and immunogold particles can be found in Supplementary Table S1. Grids were washed again six times with 0.1% BSA-c, followed by three times washing with PBS. To fix the labelled sample, the grid was incubated for 5 min with 2% glutaraldehyde (Sigma-Aldrich) and washed six times with milli-Q water. Next, the grids were transferred to 0.4% uranyl acetate in 2% methyl cellulose and incubated for 10 min. Excess solution on the grids was removed by blotting the grid at a 45° angle once from the side of the grid with filter paper. Grids were imaged using a Tecnai 12 electron microscope (FEI, Fisher Scientific) operated at 80 kV. Images were recorded at 60,000x magnification and processed using ImageJ [28]. The presence of a 6-nm gold particle on the EV surface indicated the presence of CD9 or CD63 on EV surface, whereas the presence of a 10-nm gold particle indicated the presence of BSA FITC in EV.

### 4.6. EV Isolation and Concentration

Before the EV isolation, the Amicon^®^ ultra centrifugal filter 100 kDa (Merck Millipore) was washed with EV-depleted serum-containing medium by centrifuging at 4000× *g* for 30 min. The eluate was thrown. Next, 4 mL of supernatant containing EV with CTG, BSA FITC, or Cre recombinase which was collected after USMB treatment on HUVEC cells was added to the filtration device and centrifuged at 4000× *g* for 30 min. The eluate was thrown and the filter was washed once with EV-depleted serum-containing medium followed by centrifuging at 4000× *g* for 30 min. Finally, the EV concentrate was collected and the same medium was added up to get 750 µL EV suspension. As negative controls, the supernatant containing EVs without model drug that was generated at the same USMB condition was used.

### 4.7. Uptake Assay

Prior to the uptake assay, 70,000 cells (FaDu, MDA-MB231, or T47D reporter cells) were plated in the 8-chambers Millicell^®^ EZ slides (Merck Millipore) and incubated overnight. From 750 µL EV suspension, 500 µL was added to the cells (100% concentration). The rest of this EV suspension was added to cells in another chamber (50% concentration) and 250 µL EV-depleted serum-containing medium was added to fill up the chamber. We also performed a nanoparticle tracking analysis (NTA) to estimate the concentration of EVs added for co-culturing [29] (Supplementary Procedure S1). Cells were co-cultured with EVs and measured at time points 0, 2 and 4 h using a fluorescent microscope (Nikon Ti2-U from Nikon Instruments Europe BV, Amsterdam, The Netherlands; EVOS FL from Fisher scientific). At 4 h-time point cells were washed with PBS and followed by acid wash buffer (0.5 M NaCl, 0.2 M acetic acid) to remove EVs which were on the cell surface and not taken up. Cells were washed once more with PBS before fixing with 4% paraformaldehyde (Sigma-Aldrich) or harvesting them for further analysis. For fluorescence microscopy measurement, fixed cells were stained with 50 nM LysoTraker™ Red DND-99 (LTRD) (Fisher Scientific) to stain the cell lysosomal compartment and one drop of Fluoroshield with DAPI (Sigma-Aldrich) to stain the cell nuclei; and imaged using a confocal microscope (TCS SP5 X, Leica Microsystems B.V., Amsterdam, The Netherlands). For flow cytometry measurement, cells were detached using trypsin-EDTA solution. After serum containing- medium was added, cells were pelleted by centrifuging at 250× *g* for 3 min at RT. The supernatant was carefully removed and cells were finally re-suspended in 300 µL PBS containing 0.5% BSA for flow cytometry measurement using BD FACScanto II. The uptake of CTG, BSA FITC and Cre recombinase by cells was measured at wavelength 530/30 nm.

### 4.8. RT-PCR

To detect Cre recombinase gene expression in T47D reporter cells, RT-PCR was performed. T47D receptor cells after 4 h of co-culturing with EV containing Cre recombinase were harvested. The cell pellet was dissolved in 1 mL Trizol (Fisher Scientific) per 1 million cells. The cell lysate was incubated at RT for 10–20 min. This lysate could be frozen at −20 °C prior to RNA isolation. For RNA isolation, chloroform (Sigma) was added to the cell lysate at a dilution of 1:5. The mixture was shaken vigorously for 15 s and then incubated for 2 min at RT. Next, this mixture was centrifuged for 15 min at 12,000× *g* at 4 °C. The upper aqueous phase was collected and 1 μL of GlycoBlue (Fisher Scientific) was added. Isopropanol (Merck Millipore) at a dilution of 1:2 was added to each sample and vortexed thoroughly. The sample was incubated for 10 min at RT and then centrifuged for 10 min at 12,000× *g* at 4 °C. The supernatant was carefully removed and the pellet was resuspended in 80% ethanol (Merck Millipore) at the same volume as the cell lysate. Next, the sample was centrifuged for 5 min at 7500× *g* at 4 °C. The supernatant was carefully removed and the pellet was air-dried for 10–15 min at RT. RNA was reconstituted in 20 μL nuclease-free water (Fisher Scientific) and quantified by Nanodrop (DeNovix Inc., Wilmington, DE, USA). The cDNA was prepared using Superscript 4 (Fisher Scientific) according to the manufacturer’s instructions. The cDNA was amplified using a PCR with Cre-specific primers (forward primer: 5′ GCCTGCATTACCGGTCGATGC 3′; reverse primer: 5′ GTGGCAGATGGC GCGGCAACA 3′) [15]. Thermal cycle conditions used for all reactions were as follows: 5 min at 95 °C, followed by 40 cycles consisting of denaturation for 15 s at 95 °C, annealing for 30 s at 58 °C and extension for 1 min at 72 °C. PCR reactions were concluded with incubation for 7 min at 72 °C to complete the extension of all synthesised products. PCR products were then visualised on a 1.5% TAE agarose gel (Roche Diagnostics, Manneheim, Germany).

### 4.9. Statistical Analysis

Statistical analysis was performed using GraphPad Prism 8 (La Jolla, CA, USA). ANOVA (one-way) followed by Tukey’s multiple comparison tests were used to analyse and define significant differences between samples (untreated, 0.6, 0.7 and 0.8 MPa). Differences were considered significant when *p*-values were <0.05.

## Supplementary Materials

Supplementary materials can be found at https://www.mdpi.com/1422-0067/21/8/3024/s1.
