# Supplementary material for: Potential Use of Extracellular Vesicles Generated by Microbubble-Assisted Ultrasound as Drug Nanocarriers for Cancer Treatment
Source: Int J Mol Sci. 2020 Apr 24;21(8):3024. doi: 10.3390/ijms21083024 (PMC7216118; doi:10.3390/ijms21083024)
Supplement: Supplementary file 1 [file ijms-21-03024-s001.zip › ijms-776565-supplementary/ijms-776565supplementary.docx]

**Supplementary Procedure S1**

*Nanoparticle tracking analysis*

An NS300 (Malvern Panaltical, Malvern, UK) equipped with an EMCCD camera (Andor Technology, Tokyo, Japan) and a 405-nm laser was used. During measurements, temperature was kept at 22°C. The viscosity of water at 22°C (0.952-0.953 cP) was used, as samples were diluted several folds in PBS buffer. NTA 3.2 software was used for data analysis. Before the measurement, samples were 15-30-fold diluted with PBS buffer. Six videos of 60 seconds were captured per measurement at camera level 14. The detection threshold was set at pixel value 5-13.

**Supplementary Table S1**

| **Name** | **Clone** | **Dilution** | **Manufacturer** |
| --- | --- | --- | --- |
| Alexa Fluor 647 mouse  anti-human CD9 antibody | MEM-61 | 1:200 | EXBIO Antibodies, Vestec, Czech  Republic |
| Alexa Fluor 647 mouse  anti-human CD63 | H5C6 | 1:200 | BD Pharmingen, San Jose, CA, USA |
| Alexa Fluor 647 mouse  IgG1κ isotype control | MOPC-21 | 1:200 | BD Pharmingen |
| Mouse anti-human CD9 PE |  | 1:2.5 | BD Pharmingen |
| Mouse IgG1 isotype control PE |  | 1:20 | BD Pharmingen |
| Mouse anti-human  CD63 antibody | MEM-259 | 1:10 | Abcam |
| Mouse anti-human CD9 | M-L13 | 1:50 | BD Pharmingen |
| Mouse IgG1 isotype  Control | X40 | 1:50 | BD Biosciences, San Jose, CA, USA |
| Goat anti-mouse IgG 6 nm |  | 1:50 | Aurion, Wageningen, The  Netherlands |
| Mouse anti-FITC 10 nm |  | 1:20 | Aurion, Wageningen, The  Netherlands |

**Supplementary Figures**


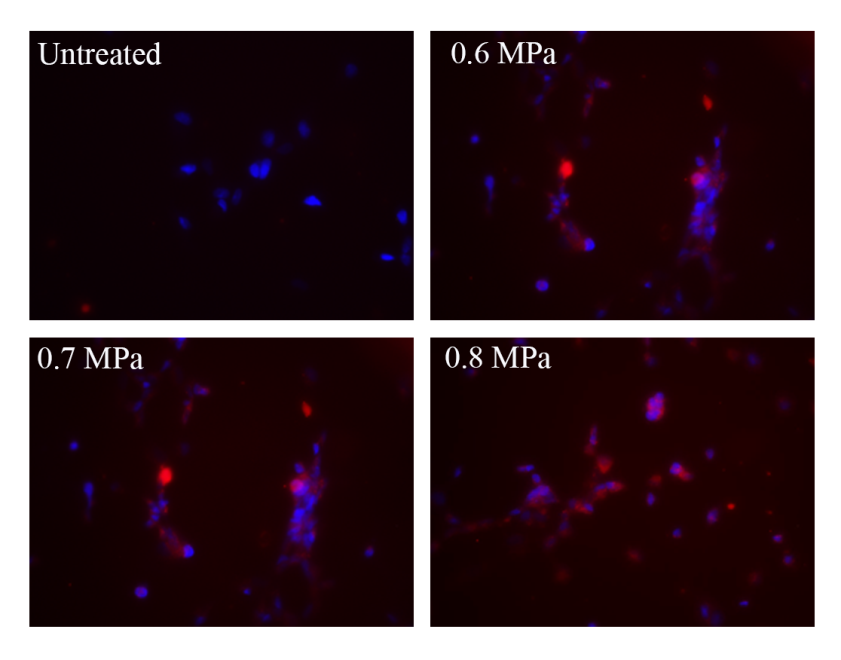


**Figure S1. Uptake of DQ-Red BSA by HUVECs at different US acoustic pressures.** HUVECs were co-administered with MB and DQ-Red BSA before application of US at 0.6, 0.7, and 0.8 MPa. The presence of bright red spots in HUVECs after this treatment indicating the presence of DQ-Red BSA in the lysosomal compartment. DAPI (blue) was used to stain cell nuclei.


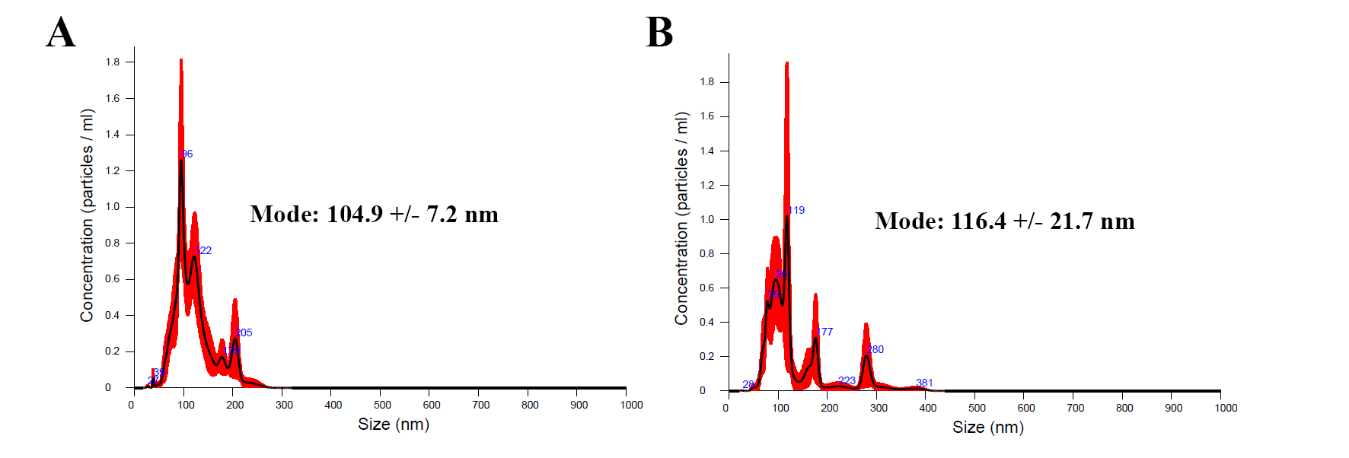


**Figure S2. Characterization of the diameter size of EVs carrying CTG and BSA FITC using NTA.** The diameter size of EVs carrying CTG is 104.9 +/-7.2 nm (**A**) and EVs carrying BSA FITC is 116 +/- 21.7 nm (**B**). EVs larger than 500 nm were not observed in both samples.


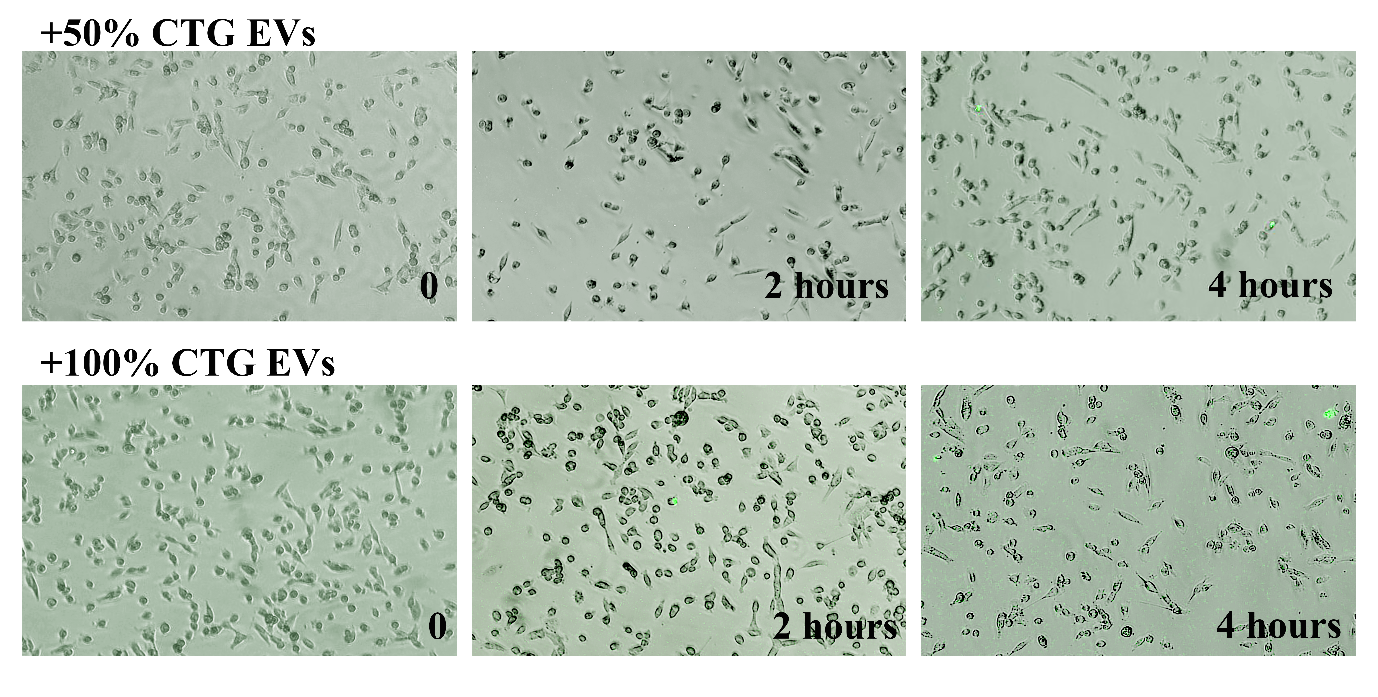


**Figure S3. Uptake of EVs containing CTG by MDA-MB-231 cells.** EVs containing CTG at a concentration of 50 % and 100 % were co-cultured with MDA-MB-231 cells and imaged at 0, 2, and 4 hours. The overlay of bright field and fluorescent images taken at 10x magnification are shown. Green fluorescent colour shown in the images indicates the presence of CTG in cells.


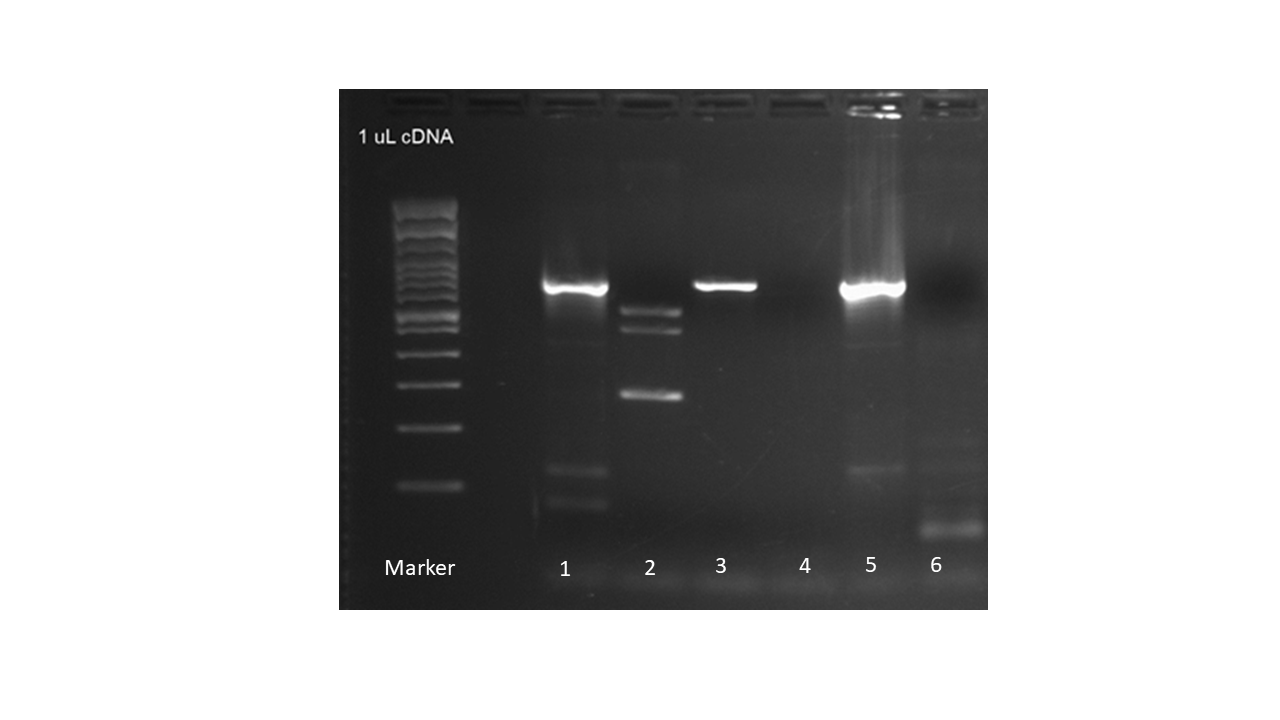


**Figure S4. Gel electrophoresis of PCR products from MDA-MB-231 cells and EVs carrying cre-recombinase.** MDA-MB-231 cells carrying cre recombinase were treated with USMB at 0.7 MPa. EVs released in the supernatant at 4 hours post treatment were harvested, RNA was extracted, and cDNA was synthesized. PCR was run using 1 µL of cDNA and the products were analyzed by gel electrophoresis. MDA-MB-231 containing cre recombinase showed a band at 760 bp which indicated the presence of cre recombinase gene (1). MDA-MB-231 cells without cre-recombinase were used as a negative control and there was no detectable cre-recombinase band at 760 bp (2). EVs extracted from the supernatant of MDA-MB-231 cre recombinase (4 hour-post USMB treatment) contained cre recombinase and there was a clear band at 760 bp (3). As a negative control, EVs from MDA-MB-231 cells were also measured and there was no cre recombinase shown (4). Second RNA extraction and DNA synthesis of MDA-MB-231 cre recombinase and MDA-MB-231 cells were performed. PCR measurements were done and results were similar as the first measurements. MDA-MB-231 cre recombinase showed a cre recombinase band at 760 bp (5), whereas MDA-MB-231 did not show this band (6).
